# Genotype affects free amino acids of egg yolk and albumen in Japanese indigenous breeds and commercial Brown layer chickens

**DOI:** 10.1016/j.psj.2021.101582

**Published:** 2021-11-07

**Authors:** Tatsuhiko Goto, Kosei Ohya, Masahiro Takaya

**Affiliations:** ⁎ Research Center for Global Agromedicine, Obihiro University of Agriculture and Veterinary Medicine, Obihiro, Hokkaido 080-8555, Japan; † Department of Life and Food Sciences, Obihiro University of Agriculture and Veterinary Medicine, Obihiro, Hokkaido 080-8555, Japan; ‡ Mie Prefecture Livestock Research Institute, Matsusaka, Mie 515-2324, Japan; § Hokkaido Tokachi Area Regional Food Processing Technology Center, Tokachi Foundation, Obihiro, Hokkaido 080-2462, Japan

**Keywords:** amino acid, breed, egg, indigenous chicken, genotype

## Abstract

Using a variety of genetic resources, the aim of this study is to see how genetic background affects egg traits in chickens. Three different chicken genotypes (a commercial Brown layer, BOR; 2 Japanese indigenous breeds, NGY and YKD) were investigated effects on genotype in 10 external and internal egg quality traits along with 20 yolk and albumen free amino acid traits. Significant effects on genotype in 10 external and internal egg quality traits and 18 yolk and 17 albumen amino acid traits were found (*P* < 0.05). In sizes and weights of egg and eggshell redness, there were significant differences among all combinations of genotype (BOR > NGY > YKD). In 14 yolk (Asn, Ser, Gln, Gly, His, Arg, Ala, Pro, Tyr, Val, Met, Leu, Phe, and Lys) and 8 albumen amino acid traits (Gln, Gly, His, Arg, Val, Ile, Leu, and Lys), BOR was significantly higher than NGY and YKD, while the opposite relations were seen in 2 amino acid traits (Cys and GABA). Moreover, phenotypic correlation analyses revealed that positive correlations among amino acid traits within each yolk and albumen were broadly seen (0.30 < r < 0.98, *P* < 0.05). However, there are almost no phenotypic correlations in amino acids between yolk and albumen in BOR and NGY, but negative correlations in YKD, which implying a potential use of untapped genetic resources for modifying amino acid balance. These results indicate genetic background affects not only sizes and weights of egg but also amino acid contents and their balance of yolk and albumen.

## INTRODUCTION

Genetic diversity is crucial for sustainable livestock industry in the world. The Hunger Map (2020) indicates prevalence of undernutrition in the population of each country and suggests that the number of hungry people will reach 840 million by 2030 if current trends of food production and population explosion continue. Moreover, since global population is likely to reach more than 9 billion by 2050, the livestock sector, the largest land user on Earth, is required to increase livestock production intensively and reduce environmental impacts (Herrero and Thornton, 2013; Michalk et al., 2019). To ensure sustainable and equitable food security, especially for feeding 9 billion people (Godfray et al., 2010), world's livestock industry has to enhance the productivity and quality of livestock products with less environmental loads by using a variety of genetic resources.

The poultry industry mainly uses long-term selected layers for egg production and broilers for meat production. Although the layer and broiler systems contribute most of global poultry production (Mottet and Tempio, 2017), they may not be sufficient varieties for all corners of the world. Mottet and Tempio (2017) mentioned the backyard production system makes significant contribution to eggs and meat production in Eastern Europe, South Asia, and sub-Saharan Africa, and smallholder farmers maintain 30 to 80% of the total poultry population in most developing countries. There are several efforts for creating crossbreds adapted to harsh production environments, in order to improve productivity of local livestock in the backyard system of developing countries (Leroy et al., 2016). As an example, Kuroiler, a new synthetic hybrid chicken introduced to Africa from India, is dual-purpose scavenger chicken for egg and meat production (Mpenda et al., 2019). Kuroilers are said to be resistant to infectious diseases and poor nutrition, and therefore can adapt under harsh tropical environmental conditions like local African chickens (Mpenda et al., 2019). Such populations, including indigenous chickens and ecotypes, fancy breeds, and experimental strains, may be valuable reservoirs of genetic diversity, which threatened by modern breeding practices (Groeneveld et al., 2010). Therefore, conservation of genetic diversity and evaluation of performance in poultry genetic resources has a key role to use natural resources efficiently, adapt to climate change, and reduce environmental impacts (Mottet and Tempio, 2017).

Japanese indigenous chickens are counted approximately 50 breeds, which are categorized as Japanese fancy breeds and Japanese utility breeds for the purposes of ornamental uses and egg and meat production, respectively (Tsudzuki, 2003). Osman et al. (2006) have reported that these Japanese indigenous chicken breeds possess clear separation in genetic background from foreign breeds such as White Leghorn and Rhode Island Red, based on the results of phylogenetic tree using 20 microsatellite DNA markers. Of them, we selected Nagoya and Yakido breeds in this study, since they are kept in the same controlled environments (such as feed materials, light control, and cage rearing system) of the Mie Prefecture Livestock Research Institute, Japan. Nagoya is one of the Japanese utility breeds and recently egg-type and meat-type Nagoya strains have established by long-term selective breeding programs (Nakamura et al., 2011). Nagoya used in the present study is a meat-type strain that derived from the Nara Prefectural Livestock Experiment Station, Japan. Yakido (Mie Game) is known as one of the Shamo classification, which includes seven breeds such as Oh-Shamo and Ko-Shamo (Tsudzuki, 2003). Yakido indicates Malay-type body shape and its main habitat is Mie Prefecture (Tsudzuki, 2003). Since Nagoya and Yakido breeds are used for mating to produce Jidori commercial chickens, their eggs are continuously available in the Livestock Research Institute. Previous works revealed that hens from the other Japanese fancy breeds, Oh-Shamo and Onagadori, produce some differences in egg characteristics in comparison with White Leghorn (Goto et al., 2015; Goto and Tsudzuki, 2017). Therefore, Japanese indigenous chickens will have a potential to express distinct feature of egg traits.

Genetic factors in egg traits, especially sizes and weights of the eggs, have been investigated well (e.g., Honkatukia et al., 2013; Wolc et al., 2014; Lue et al., 2018; Goto et al., 2019a; Qu et al., 2019). On the other hand, there are limited reports on genetic factors in constituent of egg yolk and albumen. Effects on genotype have been reported in egg contents of protein, lipid, fatty acid, and chemical composition (e.g., Onbasilar et al., 2017; Antova et al., 2019; Franco et al., 2020; Ariza et al., 2021). Recently, we have investigated free amino acids and over 130 metabolites of yolk and albumen using Rhode Island Red and Australorp breeds (Goto et al., 2019b, 2021a; Mori et al., 2020). Although amino acid, which is known to show several tastes (Kirimura et al., 1969), is one of most important food components, there are limited papers showing genetic effect on egg amino acid (Attia et al., 2020; Mori et al., 2020; Goto et al., 2021a,b). Although these previous results indicated genetic background can affect substantial differences in egg contents, further studies are needed using wide varieties of indigenous breeds in the world. In Japan, a commercial Brown layer (Boris Brown) is often used for brown egg production, but there is also limited reports showing egg amino acid contents. Untapped chicken breeds with commercial layers will have further potential to enhance egg quality from various aspects.

In this study, we analyzed 10 external and internal egg quality traits and 40 free amino acid traits of yolk and albumen using three chicken genotypes, which are 2 Japanese indigenous breeds (Nagoya and Yakido) and a commercial Brown layer (Boris Brown). In order to see how genetic background affects egg quality traits, the aim of this study is to reveal effect on genotype in comprehensive external and internal egg quality traits along with yolk and albumen free amino acid analyses in chickens.

## MATERIALS AND METHODS

### Animals and Genetic History

**Figure 1:**
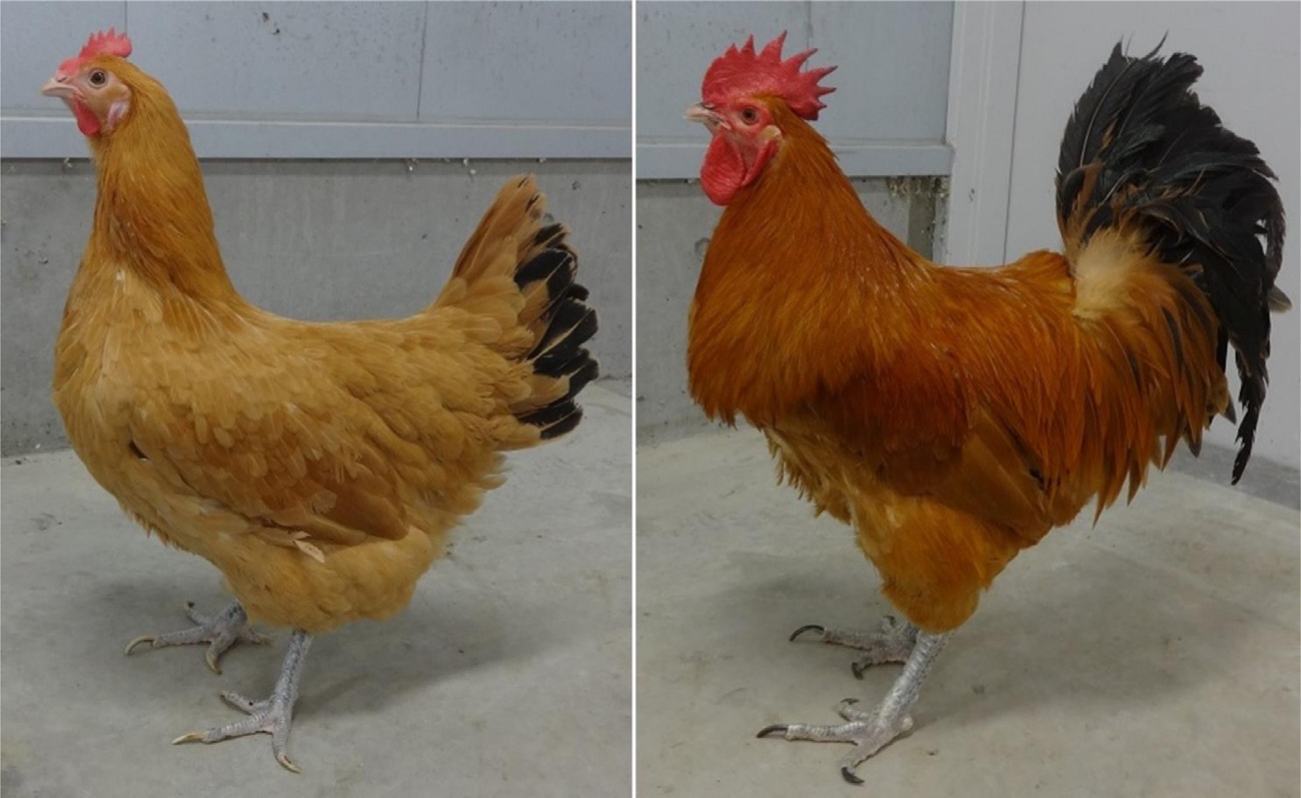
Nagoya breed. Female and male of Nagoya are shown in left and right, respectively.

**Figure 2:**
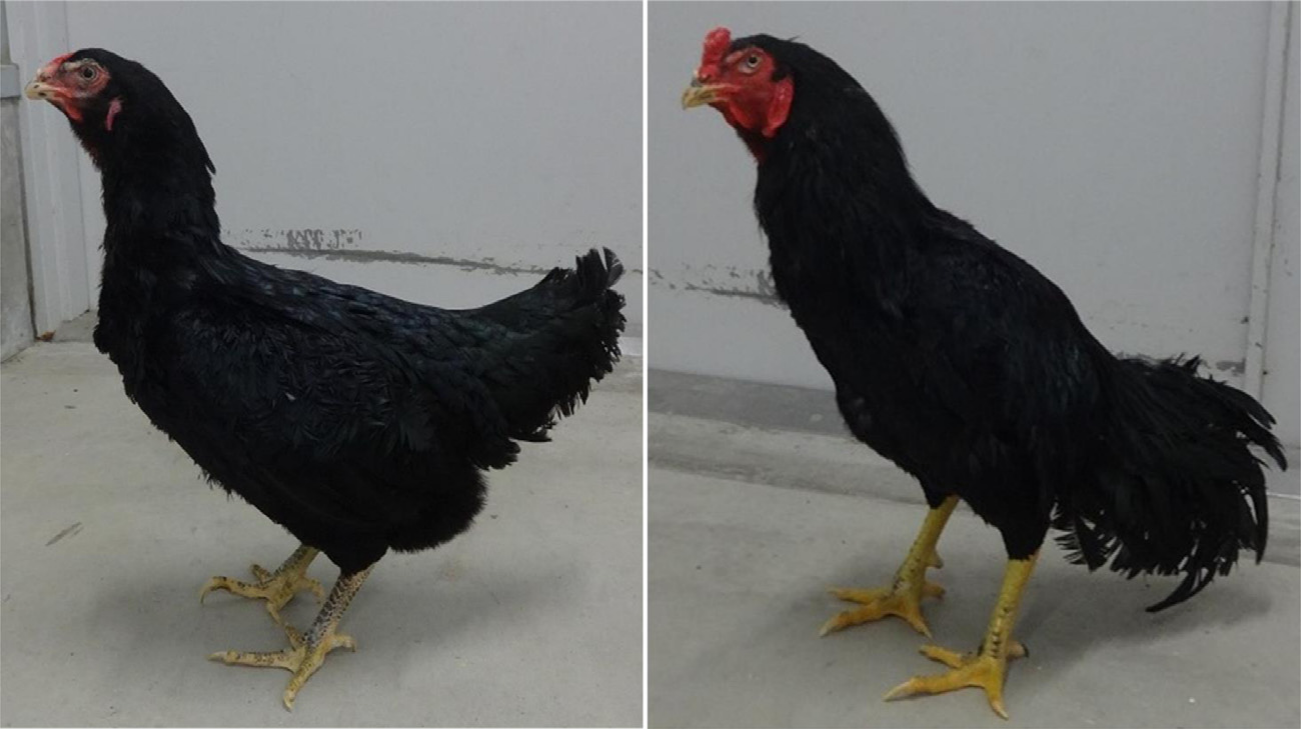
Yakido breed. Female and male of Yakido (Mie Game) are shown in left and right, respectively.

A total of 45 adult hens from three genotypes, which are a commercial Brown layer, Boris Brown (BOR; n = 15) and 2 Japanese indigenous breeds, Nagoya (Figure 1, NGY; n = 15), and Yakido (Figure 2, YKD; n = 15), were used. BOR is originally from Hy-Line International, USA via Ghen Corporation Co., Ltd., Japan. In the Mie Prefecture Livestock Research Institute, a Jidori commercial chicken, which is called Kumano-Jjidori, is created for high quality meat production. YKD breed has historically established in Mie prefecture and its meat is known to be better. Since meat-type NGY is widely accepted for Jidori commercial chickens in Japan, the stock of NGY has introduced from the Nara Prefectural Livestock Experiment Station. And therefore, these 2 breeds (NGY and YKD) are kept for parental stocks in the Mie Prefecture Livestock Research Institute.

These hens were reared in cages under the photoperiod cycle of 16 h light and 8 h dark with free access to mixed feed for layers (Pearlmash; Nojimashiryo Co., Ltd., Japan) and water in the Mie Prefecture Livestock Research Institute, Japan. Ingredient of the mixed feed (16% CP and 2,890 kcal ME kg^−1^) was shown in Table 1. These hens were kept in the same controlled environments including feed materials, light control, and cage system, in order to compare egg traits among 3 genotypes. Daily management was conducted according to the rules of Standards Related to the Care and Management of Experimental Animals. This study (authorization number 18–15) was approved from the Experimental Animal Committee of the Obihiro University of Agriculture and Veterinary Medicine.

**Table 1:** Ingredients of mixed feed.

Ingredient	Mixed feed
Crude protein (%)	16.0
Crude fat (%)	3.0
Crude ash (%)	15.0
Crude fiber (%)	6.0
Calcium (%)	3.0
Phosphate (%)	0.2
ME (kcal/kg)	2890

### Measurement of External and Internal Egg Quality Traits

An egg from each hen (total of 45 eggs) was collected at middle to late egg production stages. We measured 10 external and internal egg quality traits including egg weight (**EW**), length of the long axis of egg (**LLE**), length of the short axis of egg (**LSE**), albumen weight (**AW**), yolk weight (**YW**), eggshell weight (**SW**), eggshell thickness (**ST**), eggshell lightness (**SCL**), eggshell redness (**SCR**), and eggshell yellowness (**SCY**). Weights were measured with an electronic balance (EK-6000H; A&D Company, Ltd, Japan), whereas sizes and eggshell thickness were measured using a digital caliper (P01 110–120; ASONE, Japan) and a Peacock dial pipe gauge P-1 (Ozaki MFG Co, Ltd, Japan), respectively. Eggshell colors (lightness, redness, and yellowness in the L*a*b* color spaces) were measured using a chromameter (CR-10 Plus Color Reader; Konica Minolta Japan, Inc, Japan). A previous report (Dunn et al., 2019) confirmed that breeders can use Minolta colorimetry with confidence to assess differences in staining to estimate cuticle deposition (absorbance at 640 nm). After measuring yolk weight, the yolk was diluted 2-fold with distilled water. Yolk solution and albumen were kept in each tube at –30°C until use.

### Yolk and Albumen Amino Acid Analyses

Amino acid analysis for yolk was performed following the previous method (Mori et al., 2020). Yolk solution (5 mL) was mixed with 5 mL of 16% trichloroacetic acid solution (FUJIFILM Wako Chemicals, Japan) and vortexed. The samples were centrifuged at 1,400 × *g* for 15 min using a centrifuge (RX II series; HITACHI Ltd., Japan). The supernatant was collected using a syringe (NIPRO Corporation, Japan) and then filtered out a microtube through a disposable cellulose acetate membrane filter unit with a 0.45-μm pore size (DISMIC-25CS; Advantec Toyo Kaisha, Ltd, Japan). For albumen, 250 μL of the sample (mixture of thin albumen and thick albumen) was mixed with 250 μL of 16% trichloroacetic acid solution. After vortexing, the sample was centrifuged at 11,000 × *g* for 15 min. The supernatant was filtered as well as the yolk sample.

The filtered solution (40 μL) was dried at 40°C for 90 min using a vacuum oven (VOS-201SD, Eyela, Japan). After adding 20 μL of mixing solution (ethanol: DW: triethylamine = 2:2:1), the microtube was vortexed for 20 min using a Micro Mixer E-36 (TAITEC Corporation, Japan). The sample was dried at 40°C for 60 min in a vacuum oven. The 20 μL of mixing solution (ethanol: DW: triethylamine: phenylisothiocyanate = 7:1:1:1) was added and mixed for 20 min using the Micro Mixer. The sample was heated at 40°C for 60 min in a vacuum oven for drying. After preprocessing, the sample tube was kept at –30°C until the sample was analyzed.

Yolk and albumen free amino acids were analyzed by HPLC (LC-2010CHT; Shimadzu Co. Ltd., Japan). Amino acid standards (types H and B), L-asparagine, and L-glutamine (FUJIFILM Wako Chemicals, Japan) were prepared following the same method for the sample preprocessing. The standards were analyzed before every 30 samples. The absolute concentration was calculated from the peaks of sample and standard. In this study, 20 kinds of amino acids (Asp, Glu, Asn, Ser, Gln, Gly, His, Arg, Thr, Ala, Pro, GABA, Tyr, Val, Met, Cys, Ile, Leu, Phe, and Lys) were measured in yolk and albumen, and therefore trait names were prefixed as Y_ and A_ for yolk and albumen amino acids, respectively.

### Statistical Analyses

To test the effect on genotype in external and internal egg quality traits and yolk and albumen amino acid traits, data were analyzed using one-way analysis of variance (**ANOVA**) with a significant threshold (*P* < 0.05). Tukey's honestly significant difference (**HSD**) test was performed to check inter-genotype differences (*P* < 0.05). Shapiro-Wilk test and non-parametric Kruskal-Wallis rank sum test were used to test normality and effect on genotype, respectively. Data are shown as mean and standard deviation. Statistical analyses were conducted using R (R Core Team, 2020). Pearson's correlation coefficients among all traits were calculated and plotted using ‘corrplot’ package of R. The significant threshold (*P* < 0.05) was used to check whether correlation coefficients equal zero or not.

## RESULTS

All traits were checked whether the distribution fits normal distribution or not via Shapiro-Wilk normality test. Almost all traits fitted normal distribution (*P* > 0.05). Some of them (e.g., Y_GABA) were non normal distribution (*P* < 0.05), because there were a lot of zero values in Y_GABA. In order to enhance the robustness, we conducted non-parametric Kruskal-Wallis rank sum tests for all traits. As shown in Supplementary Table 1, ANOVA results were supported by Kruskal-Wallis test. Therefore, we mentioned ANOVA results only in this paper.

### External and Internal Egg Quality Traits

Table 2 indicated mean and standard deviation of 10 kinds of external and internal egg quality traits. One-way ANOVA revealed significant effects on genotype in all traits (*P* < 0.001). In sizes and weights (egg weight, length of the long axis of egg, length of the short axis of egg, albumen weight, and eggshell weight), all combinations among genotype were significantly different by Tukey's HSD tests. BOR and YKD were the highest and lowest, whereas NGY was intermediate. While BOR was significantly higher than NGY and YKD in YW, YKD was significantly lower than BOR and NGY in eggshell thickness. In eggshell color, BOR was significantly lower and higher in lightness and yellowness than NGY and YKD, respectively. In eggshell redness, there were significant differences among all combinations (BOR > NGY > YKD).

**Table 2:** Mean and standard deviation of external and internal egg quality traits in three genotypes of chickens.

Trait^1^	Boris Brown	Nagoya	Yakido	One-way ANOVA^2^			
	(BOR; *n* = 15)	(NGY; *n* = 15)	(YKD; *n* = 15)	df_bet_	df_res_	F value	*P* value
EW (g)	61.3 ± 4.7^a^	55.3 ± 2.7^b^	45.0 ± 2.8^c^	2	42	82.2	3.0E-15⁎⁎⁎
LLE (mm)	57.9 ± 2.2^a^	56.3 ± 1.8^b^	51.9± 1.0^c^	2	42	50.1	7.6E-12⁎⁎⁎
LSE (mm)	43.6 ± 1.2^a^	42.1 ± 0.8^b^	39.8 ± 1.1^c^	2	42	49.1	1.0E-11⁎⁎⁎
AW (g)	36.1 ± 3.5^a^	32.6 ± 2.4^b^	24.7 ± 1.5^c^	2	42	75.7	1.2E-14⁎⁎⁎
YW (g)	17.5 ± 1.6^a^	15.9 ± 0.9^b^	15.0 ± 1.5^b^	2	42	13.5	3.0E-05⁎⁎⁎
SW (g)	7.5 ± 0.6^a^	6.6 ± 0.5^b^	5.1 ± 0.6^c^	2	42	65.9	1.1E-13⁎⁎⁎
ST (mm)	0.44 ± 0.03^a^	0.45 ± 0.03^a^	0.37 ± 0.03^b^	2	42	35.8	8.4E-10⁎⁎⁎
SCL	57.0 ± 3.1^b^	76.0 ± 4.0^a^	77.3 ± 3.0^a^	2	42	169.8	2.2E-16⁎⁎⁎
SCR	18.9 ± 1.8^a^	8.5 ± 2.4^b^	5.6 ± 1.8^c^	2	42	182.4	2.2E-16⁎⁎⁎
SCY	26.8 ± 0.9^a^	15.5 ± 2.6^b^	17.2 ± 3.0^b^	2	42	102.7	2.2E-16⁎⁎⁎

1 AW, albumen weight; EW, egg weight; LLE, length of the long axis of egg; LSE, length of the short axis of egg; SCL, eggshell color lightness; SCR, eggshell color redness; SCY, eggshell color yellowness; SW, eggshell weight; ST, eggshell thickness; YW, yolk weight.

a,b,c Means with different superscripts differ significantly among genotype (*P* < 0.05).

2 df_bet_: between groups degree of freedom, df_res_: residual degree of freedom,

⁎⁎⁎ *P* < 0.001.

### Yolk Amino Acid Traits

One-way ANOVA indicated significant effects on genotype (*P* < 0.05) in 18 yolk amino acid traits (Y_Asp, Y_Glu, Y_Asn, Y_Ser, Y_Gln, Y_Gly, Y_His, Y_Arg, Y_Thr, Y_Ala, Y_Pro, Y_Tyr, Y_Val, Y_Met, Y_Cys, Y_Leu, Y_Phe, and Y_Lys), except for Y_GABA and Y_Ile (Table 3). Of the 18 traits, BOR was significantly higher contents than NGY and YKD in 14 yolk amino acid traits (Y_Asn, Y_Ser, Y_Gln, Y_Gly, Y_His, Y_Arg, Y_Ala, Y_Pro, Y_Tyr, Y_Val, Y_Met, Y_Leu, Y_Phe, and Y_Lys) by Tukey's HSD tests. BOR was significantly higher than NGY in 2 traits (Y_Asp and Y_Thr), whereas YKD was significantly higher than NGY in Y_Glu. YKD and NGY were significantly higher contents than BOR in Y_Cys,

**Table 3:** Mean and standard deviation of yolk free amino acid traits in three genotypes of chickens.

Trait^1^	Boris Brown	Nagoya	Yakido	One-way ANOVA^2^			
(µg/mL)	(BOR; *n* = 15)	(NGY; *n* = 15)	(YKD; *n* = 15)	df_bet_	df_res_	F value	*P* value
Y_Asp	14.9 ± 4.3^a^	10.8 ± 2.8^b^	13.1 ± 3.0a,b	2	42	5.4	0.008⁎⁎
Y_Glu	65.2 ± 10.3a,b	57.1 ±7.9^b^	66.4 ± 9.0^a^	2	42	4.5	0.016*
Y_Asn	21.1 ± 2.4^a^	15.8 ± 1.4^b^	16.1 ± 1.9^b^	2	42	35.6	9.1E-10⁎⁎⁎
Y_Ser	36.6 ± 3.5^a^	30.8 ± 2.5^b^	30.0 ± 3.4^b^	2	42	18.9	1.4E-06⁎⁎⁎
Y_Gln	38.7 ± 3.3^a^	32.8 ± 2.6^b^	32.6 ± 3.6^b^	2	42	17.5	3.0E-06⁎⁎⁎
Y_Gly	13.9 ± 1.4^a^	11.0 ± 0.9^b^	10.5 ± 1.2^b^	2	42	36.6	6.3E-10⁎⁎⁎
Y_His	11.0 ± 1.4^a^	7.6 ± 1.3^b^	7.0 ± 1.1^b^	2	42	47.2	1.8E-11⁎⁎⁎
Y_Arg	47.4 ± 4.7^a^	37.2 ± 3.3^b^	35.8 ± 4.4^b^	2	42	34.8	1.2E-09⁎⁎⁎
Y_Thr	36.9 ± 3.7^a^	29.6 ± 3.5^b^	34.3 ± 8.1a,b	2	42	6.8	0.003⁎⁎
Y_Ala	21.3 ± 2.5^a^	15.0 ± 1.5^b^	16.7 ± 1.8^b^	2	42	40.3	1.7E-10⁎⁎⁎
Y_Pro	21.9 ± 1.9^a^	18.4 ± 1.8^b^	17.4 ± 1.9^b^	2	42	24.6	8.7E-08⁎⁎⁎
Y_GABA	0.07 ± 0.14	0.03 ± 0.10	0.00 ± 0.00	2	42	1.7	0.203^ns^
Y_Tyr	37.4 ± 2.7^a^	31.3 ± 3.2^b^	30.2 ± 2.6^b^	2	42	27.8	2.0E-08⁎⁎⁎
Y_Val	32.4 ± 3.0^a^	24.7 ± 2.2^b^	26.9 ± 2.7^b^	2	42	33.7	1.9E-09⁎⁎⁎
Y_Met	16.9 ± 1.7^a^	12.1 ± 1.1^b^	12.8 ± 1.3^b^	2	42	52.4	3.9E-12⁎⁎⁎
Y_Cys	1.8 ± 0.2^b^	2.4 ± 0.2^a^	2.6 ± 0.3^a^	2	42	41.6	1.1E-10⁎⁎⁎
Y_Ile	27.2 ± 2.6	26.6 ± 2.2	27.0 ± 2.8	2	42	0.2	0.792^ns^
Y_Leu	56.0 ± 4.6^a^	42.8 ± 3.8^b^	44.9 ± 4.9^b^	2	42	37.7	4.3E-10⁎⁎⁎
Y_Phe	31.8 ± 2.6^a^	22.0 ± 1.9^b^	23.9 ± 2.3^b^	2	42	76.8	9.2E-15⁎⁎⁎
Y_Lys	56.3 ± 5.7^a^	41.4 ± 4.0^b^	42.0 ± 5.1^b^	2	42	43.4	5.9E-11⁎⁎⁎

1 Trait abbreviations are shown in Materials and Methods. Concentration of yolk solution (2-fold diluted with DW).

a,b,c Means with different superscripts differ significantly among genotype (*P* < 0.05).

2 df_bet_: between groups degree of freedom, df_res_: residual degree of freedom,

⁎⁎⁎ *P* < 0.001.

⁎⁎ *P* < 0.01.

⁎ *P* < 0.05.

ns *P* > 0.05.

### Albumen Amino Acid Traits

One-way ANOVA revealed significant effects on genotype (*P* < 0.05) in 17 albumen amino acid traits (A_Asp, A_Glu, A_Asn, A_Ser, A_Gln, A_Gly, A_His, A_Arg, A_Ala, A_GABA, A_Val, A_Met, A_Cys, A_Ile, A_Leu, A_Phe, and A_Lys), except for A_Thr, A_Pro, and A_Tyr (Table 4). Of the 17 traits, BOR was significantly higher contents than NGY and YKD in 8 albumen amino acid traits (A_Gln, A_Gly, A_His, A_Arg, A_Val, A_Ile, A_Leu, and A_Lys) by Tukey's HSD tests. BOR and YKD were significantly higher than NGY in A_Asp, A_Glu, and A_Phe. BOR was significantly lower than YKD and NGY in A_GABA. BOR was significantly higher than NGY in 2 traits (A_Ser and A_Ala) and YKD in A_Met, respectively. YKD was significantly higher than NGY in A_Cys, while NGY was significantly higher than YKD in A_Asn.

**Table 4:** Mean and standard deviation of albumen free amino acid traits in three genotypes of chickens.

Trait^1^	Boris Brown	Nagoya	Yakido	One-way ANOVA^2^			
(µg/mL)	(BOR; *n* = 15)	(NGY; *n* = 15)	(YKD; *n* = 15)	df_bet_	df_res_	F value	*P* value
A_Asp	0.74 ± 0.25^a^	0.39 ± 0.13^b^	0.64 ± 0.19^a^	2	42	12.4	5.8E-05⁎⁎⁎
A_Glu	1.57 ± 0.53^a^	1.11 ± 0.36^b^	1.62 ± 0.45^a^	2	42	5.7	0.006⁎⁎
A_Asn	0.16 ± 0.02a,b	0.18 ± 0.03^a^	0.15 ± 0.02^b^	2	42	6.2	0.004⁎⁎
A_Ser	0.82 ± 0.34^a^	0.55 ± 0.25^b^	0.67 ± 0.22a,b	2	42	3.6	0.037*
A_Gln	0.45 ± 0.14^a^	0.30 ± 0.08^b^	0.29 ± 0.12^b^	2	42	9.2	4.8E-04⁎⁎⁎
A_Gly	0.32 ± 0.11^a^	0.17 ± 0.10^b^	0.22 ± 0.06^b^	2	42	10.6	1.9E-04⁎⁎⁎
A_His	0.54 ± 0.07^a^	0.33 ± 0.07^b^	0.31 ± 0.05^b^	2	42	58.0	8.2E-13⁎⁎⁎
A_Arg	1.36 ± 0.26^a^	0.87 ± 0.21^b^	0.97 ± 0.20^b^	2	42	20.6	5.7E-07⁎⁎⁎
A_Thr	0.74 ± 0.27	0.55 ± 0.22	0.73 ± 0.21	2	42	3.1	0.055^ns^
A_Ala	0.57 ± 0.22^a^	0.34 ± 0.16^b^	0.46 ± 0.16a,b	2	42	6.1	0.005⁎⁎
A_Pro	1.03 ± 0.42	0.76 ± 0.34	0.87 ± 0.24	2	42	2.5	0.097^ns^
A_GABA	0.17 ± 0.10^b^	0.46 ± 0.16^a^	0.46 ± 0.10^a^	2	42	29.7	9.2E-09⁎⁎⁎
A_Tyr	1.51 ± 0.33	1.54 ± 0.28	1.56 ± 0.24	2	42	0.1	0.871^ns^
A_Val	1.17 ± 0.38^a^	0.60 ± 0.21^b^	0.70 ± 0.18^b^	2	42	19.0	1.3E-06⁎⁎⁎
A_Met	3.79 ± 0.50^a^	3.63 ± 0.54a,b	3.27 ± 0.34^b^	2	42	4.8	0.013*
A_Cys	1.32 ± 0.10a,b	1.19 ± 0.05^b^	1.87 ± 1.13^a^	2	42	4.5	0.017*
A_Ile	0.88 ± 0.26^a^	0.58 ± 0.19^b^	0.64 ± 0.18^b^	2	42	8.3	9.1E-04⁎⁎⁎
A_Leu	2.72 ± 0.49^a^	2.03 ± 0.37^b^	2.07 ± 0.33^b^	2	42	14.0	2.2E-05⁎⁎⁎
A_Phe	2.48 ± 0.27^a^	2.09 ± 0.32^b^	2.44 ± 0.46^a^	2	42	5.4	0.008⁎⁎
A_Lys	0.21 ± 0.06^a^	0.10 ± 0.03^b^	0.11 ± 0.03^b^	2	42	35.8	8.5E-10⁎⁎⁎

1 Trait abbreviations are shown in Materials and Methods.

a,b,c Means with different superscripts differ significantly among genotype (*P* < 0.05).

2 df_bet_: between groups degree of freedom, df_res_: residual degree of freedom,

⁎⁎⁎ *P* < 0.001.

⁎⁎ *P* < 0.01.

⁎ *P* < 0.05.

ns *P* > 0.05.

### Phenotypic Correlations

**Figure 3:**
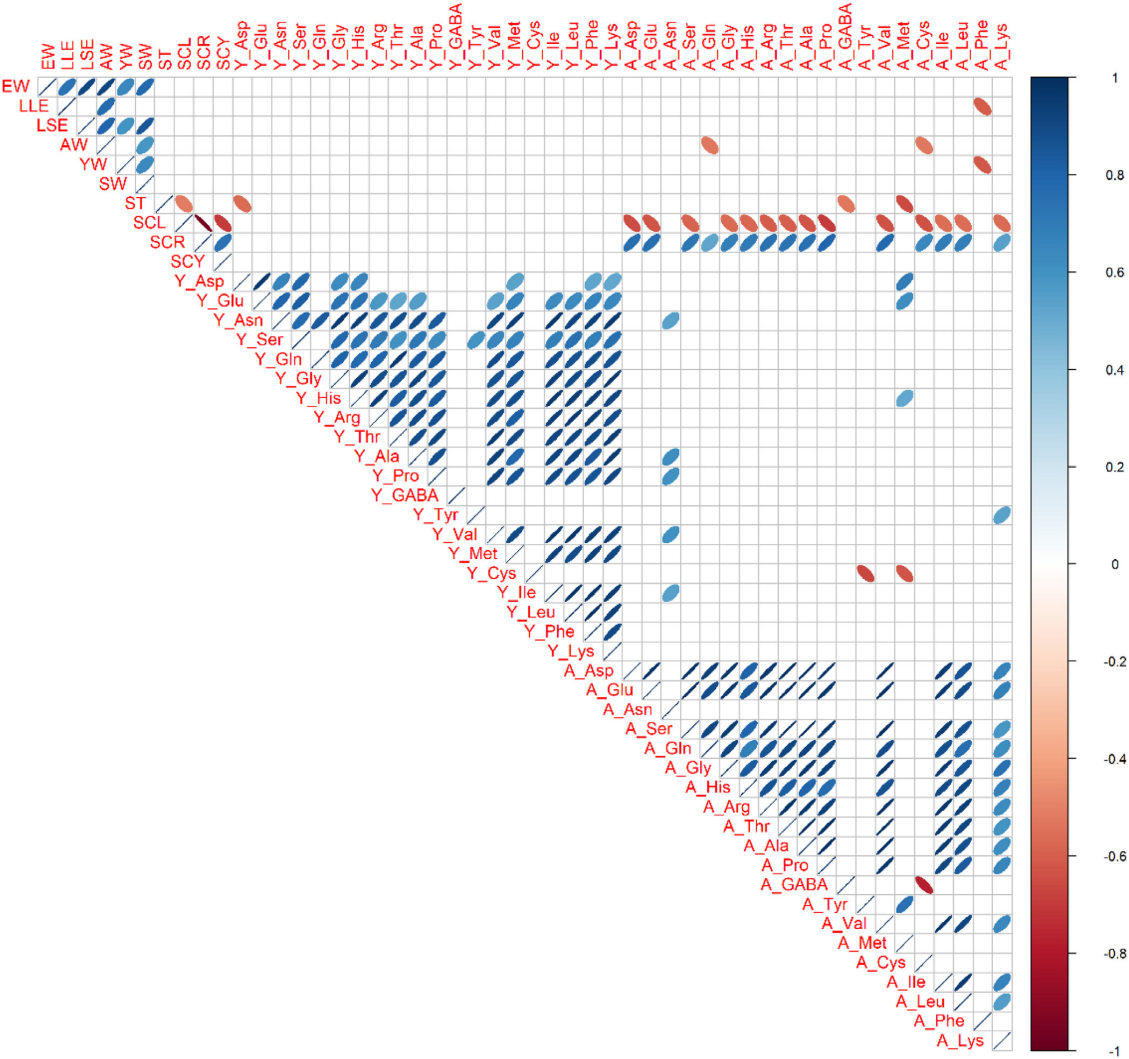
Phenotypic correlations among egg traits in Boris Brown. Ten egg traits, 20 yolk amino acids traits, and 20 albumen amino acids traits in Boris Brown (BOR; n = 15) were used. Trait abbreviations are expressed in Materials and Methods. Pearson's correlation coefficients are expressed by ellipse. Blue and red ellipse indicate positive and negative correlations in each trait pair (*P* < 0.05). Blank cells show not significance.

**Figure 4:**
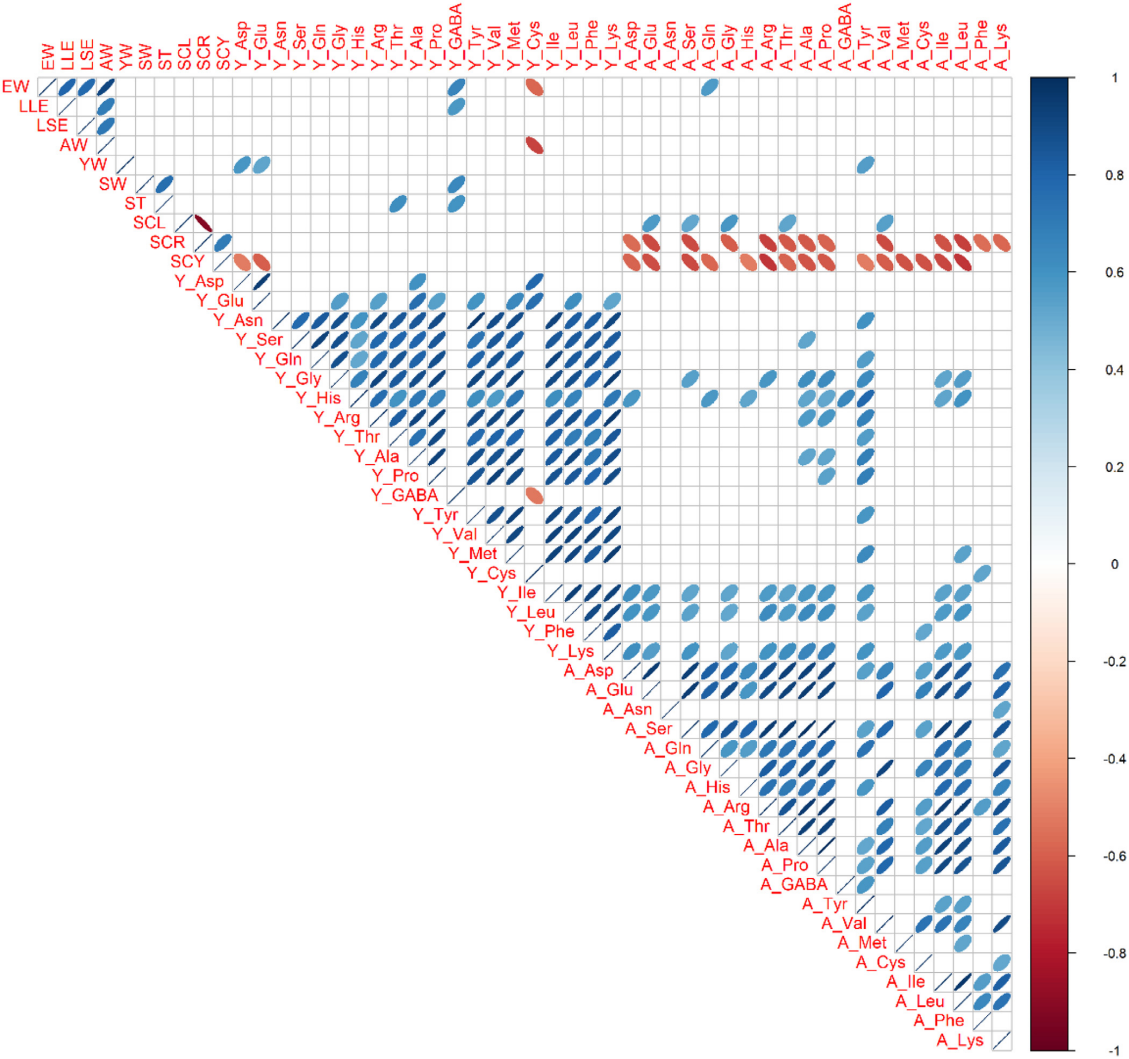
Phenotypic correlations among egg traits in Nagoya. Ten egg traits, 20 yolk amino acids traits, and 20 albumen amino acids traits in Nagoya (NGY; n = 15) were used. Trait abbreviations are expressed in Materials and Methods. Pearson's correlation coefficients are expressed by ellipse. Blue and red ellipse indicate positive and negative correlations in each trait pair (*P* < 0.05). Blank cells show not significance.

**Figure 5:**
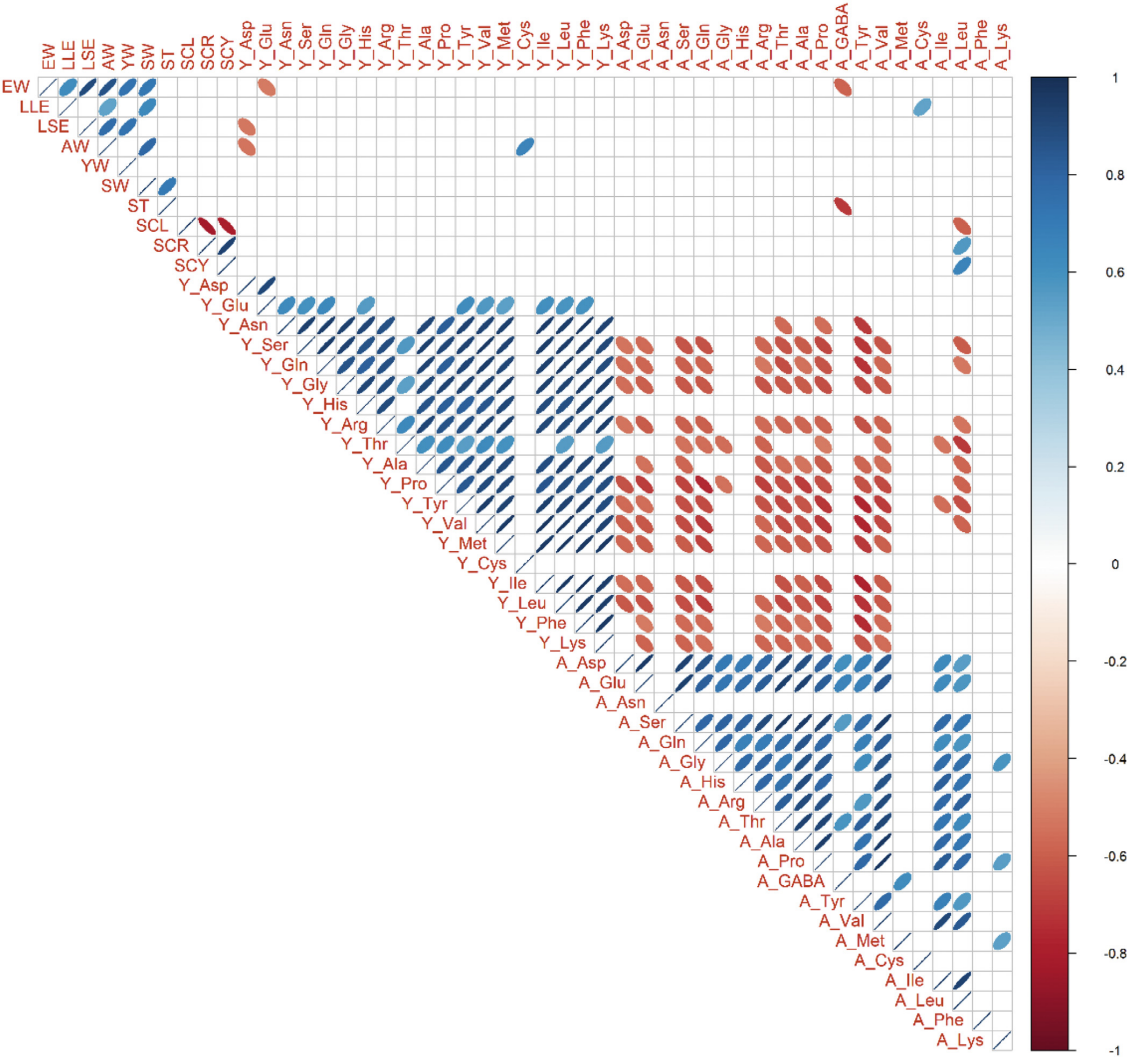
Phenotypic correlations among egg traits in Yakido. Ten egg traits, 19 yolk amino acids traits, and 20 albumen amino acids traits in Yakido (YKD; n = 15) were used. Y_GABA is excluded because of zero in all individuals of YKD. Trait abbreviations are expressed in Materials and Methods. Pearson's correlation coefficients are expressed by ellipse. Blue and red ellipse indicate positive and negative correlations in each trait pair (*P* < 0.05). Blank cells show not significance.

Phenotypic correlation coefficients were calculated among 10 external and internal egg quality traits, 20 yolk amino acids traits, and 20 albumen amino acids traits in each genotype (Figure 3, Figure 4, Figure 5). Among sizes and weights traits, there were tendencies of positive correlations (0.31 < r < 0.98, *P* < 0.05). Within each yolk and albumen, positive phenotypic correlations were broadly observed in most amino acids combinations (0.30 < r < 0.98, *P* < 0.05). On the other hand, there were almost no phenotypic correlations between yolk amino acids and albumen amino acids in BOR and NGY (Figures 3 and 4). Especially in YKD (Figure 5), negative phenotypic correlations were found broadly between yolk and albumen amino acids (−0.75 < r < −0.10, *P* < 0.05). In BOR and NGY but not in YKD, there were phenotypic correlations between eggshell colors and albumen amino acids.

## DISCUSSION

To reveal effect on genotype in external and internal egg quality traits and yolk and albumen free amino acid traits, three different chicken genotypes (BOR, NGY, and YKD) were investigated. This study found significant effects on genotype in 10 external and internal egg quality traits, 18 yolk amino acid traits and 17 albumen amino acid traits. In sizes and weights of the egg and eggshell redness, there were significant differences among all combinations of genotype (BOR > NGY > YKD). In 14 yolk amino acid traits and 8 albumen amino acid traits, BOR was significantly higher than NGY and YKD, while the opposite relations (BOR < NGY = YKD) were seen in 2 amino acid traits. Moreover, phenotypic correlations among all egg traits revealed that positive correlations among amino acid traits within each yolk and albumen were broadly seen in all 3 genotypes. However, there are almost no phenotypic correlations in free amino acids between yolk and albumen in BOR and NGY, but negative correlations in YKD. These novel findings will lead to a change in the amino acid balance of yolk and albumen by crossbreeding using YKD as a parent. Taken together, the present results clearly indicate that variation of genetic background broadly affects not only sizes and weights of egg but also free amino acid contents and balance of yolk and albumen.

This study revealed that significant differences among all combinations of genotype were seen as BOR > NGY > YKD in sizes and weights of the egg (EW, LLE, LSE, AW, and SW). The relation of avian egg weight to body weight is known in more than 800 species of birds including Galliformes (Rahn et al., 1975). Average body weight of BOR female is 1.6 to 2.1 kg according to the standards of rearing for Boris Brown (Ghen Corporation Co., Ltd., Japan), while females in NGY and YKD in the Mie Prefecture Livestock Research Institute are 2.3 to 2.6 kg and 1.7 to 2.1 kg in average body weight, respectively (Ohya, personal communications). The present results of average egg weight were 61.3 g in BOR, 55.3 g in NGY, and 45.0 g in YKD. Nakamura et al. (2011) reported that Nagoya female in 2.5 kg body weight lays 57 g egg weight in average, which supporting our results. It is reported that females in Oh-Shamo (**SHA**) around 3.0 kg and CB strain of White Leghorn (**WL**) around 1.5 kg body weight produce 53 to 55 g and 47 to 48 g egg weight, respectively (Goto et al., 2014, 2019c). These results imply that females with the smaller (CB strain of WL and YKD) and larger (NGY and SHA) body weight tend to lay smaller and larger eggs, respectively. Although this relation is widely seen in avian species (Rahn et al., 1975), BOR was not the case. Mori et al. (2020) previously reported Australorp female was the similar case as well as BOR females, which produce eggs larger than expected based upon body size. The outliers of breed and commercial layers may be likely to be created by long-term selective breeding during the classical breed formation and recent industrial strain establishment.

BOR was significantly higher contents than NGY and YKD in 14 yolk amino acid traits (Y_Asn, Y_Ser, Y_Gln, Y_Gly, Y_His, Y_Arg, Y_Ala, Y_Pro, Y_Tyr, Y_Val, Y_Met, Y_Leu, Y_Phe, and Y_Lys) and 8 albumen amino acid traits (A_Gln, A_Gly, A_His, A_Arg, A_Val, A_Ile, A_Leu, and A_Lys). It is well known that amino acids indicate several tastes (Kirimura et al., 1969). And also, we previously reported significant breed and feed effects on amino acid and taste sensor traits in egg albumen and yolk (Goto et al., 2021a). Therefore, higher contents of amino acids in BOR may have some differences in egg taste traits, which may lead to being benefits for consumers and producers by making taste-added designer eggs. BOR is made from a cross of Rhode Island Red and White Plymouth Rock. Generally speaking, since hybrids express higher vigor and productivity, the hybrids are frequently used in the livestock industry. This study revealed a potential of some benefits from heterosis in egg yolk and albumen quality in molecular levels. To study heterosis effect on free amino acids, 3 populations based on 2 parental breeds and F_1_ hybrid should be investigated. In Japan, there are many Jidori brand (hybrid chickens) based on the crosses between Japanese indigenous breeds (e.g., Hinaidori and Shamo) and Western breeds (e.g., Rhode Island Red and White Plymouth Rock). Goto et al. (2021b) have identified a significant heterosis effect on yolk aspartic acid. Therefore, further studies on heterosis effect of egg contents will be needed to know the better combinations for increasing egg quality in molecular level.

Historically, Japanese fancy breeds (including YKD) have been established for ornamental purposes, for example, for enjoying beautiful long tail feathers and saddle hackles and prolonged crowing of males, but not egg and meat production (Tsudzuki, 2003; Goto et al., 2015). Since NGY has established as Japanese utility breed for egg and meat production, there are egg-type and meat-type strains in NGY breed (Nakamura et al., 2011). Although we speculated that the recent selective breeding will enhance egg quality traits in NGY, this study cannot find any amino acid traits, which are significantly higher contents in NGY than two genotypes. The similar tendency is shown in our previous report, which indicates that egg amino acid contents from a different stock of NGY are intermediate or low in comparison with four breeds and 2 F_1_ hybrids (Goto et al., 2021b). Since meat-type Nagoya is often used for meat production in Jidori brands established by several Prefectural livestock centers in Japan, we used the meat-type NGY in the present and previous studies (Goto et al., 2021b). Since several lines of egg-type NGY are uniquely established by long-term selective breeding in Aichi Prefectural livestock center (Nakamura et al., 2011), we should analyze egg amino acid traits using any egg-type NGY in the future. On the other hand, we could find better performance of YKD in some traits of albumen free amino acids (A_Asp, A_Glu, and A_Phe), which are comparable to Brown layer. Although there are no or weak correlations between yolk and albumen amino acids in many breeds, the distinct feature of YKD was found in the negative correlations between yolk and albumen amino acids, which implying a potential use of untapped genetic resources especially YKD to modify the balance of amino acid contents in yolk and albumen. In comparison with our previous paper (Goto et al., 2021b), this study firstly found the unique feature of phenotypic correlations in YKD. Since we cannot rule out the reason why YKD is so unique, further genetic analyses are needed. Since each genotype has many variants in each genetic background, future population genomics will reveal genotype-specific unique variants throughout the genome (Goto and Tsudzuki, 2017). Of them, some variants will contribute to the differences in the balance of yolk and albumen amino acids. Therefore, further mating experiments using YKD are needed to understand the genotype-phenotype relationship. In addition, Japanese fancy chicken breeds (Ukokkei and Shamo) indicated higher contents of egg amino acids (Goto et al., 2021b). These results suggested that indigenous breeds will have potential to show better performance in some egg quality traits, regardless of the purposes in breed establishment. Therefore, future genomics studies will reveal some genetic loci affecting contents of egg amino acids using these genetic resources.

Results from phenotypic analysis in this study must be the first step not only to conduct further cross experiments among indigenous breeds for checking the performance of the offspring but also to find candidate genes affecting yolk and albumen amino acids via further genomics studies. To improve the productivity of local chickens in the backyard system of developing countries, continuous efforts have been conducted for creating crossbreds adapted to harsh production environments (Leroy et al., 2016). One of the advantages of using the world's indigenous chickens rather than modern layers will be to understand the genetic basis of egg quality traits as well as adaptive traits in the backyard production system, which will be the key to the future world livestock production. Genomics using a variety of indigenous chicken breeds have a large potential to find genetic variants underlying adaptation to each rearing environment (Lawal and Hanotte, 2021). Actually, recent population genomics using large samples of African indigenous chickens enable us to know some drivers of local adaptation (Gheyas et al., 2021). To cope with the future world food crisis (Godfray et al., 2010), it is important to reveal hidden adaptation genes toward every corner of the world via genomics using a wide variety of indigenous chickens in the world. Therefore, it is important to keep revealing unique phenotypic features of egg amino acid traits in untapped indigenous breeds along with genomics.

This study indicated that Brown layer is likely to be the best performance under the conventional cage system and corn-based mixed feed. Egg traits are well known to be affected by both genetic and environmental factors (Goto and Tsudzuki, 2017). Because a large amount of corn, which is major content of the mixed feed, has been imported in Japan (Kaku et al., 2004), feed materials should be partially replaced from the imported corn to locally available materials based on the local production and consumption. Goto et al. (2019b, 2021a) and Mori et al. (2020) have introduced that amino acids and metabolites of yolk and albumen can be altered by a fermented feed for layers, which is created from feed materials that are 100% available in Japan. In addition, recent rearing environments for layers are varied from a concept of animal welfare. Egg traits are likely to be affected by rearing systems, which include conventional cage, furnished cage, aviary, and free-range (Shimmura et al., 2010). Nishimura et al. (2021) actually have reported better performance of NGY in yolk and albumen amino acids among 5 chicken genotypes under floor rearing system, although lower egg amino acid contents of NGY were commonly seen under the cage rearing system among 3 genotypes in this study and 7 genotypes in the previous study (Goto et al., 2021b). Since environmental factors (feed materials and rearing systems) also affect egg quality traits, several indigenous chicken breeds should be tested in future under varieties of environmental conditions, which will reveal novel findings about genetic by environment interaction (Bekele et al., 2009). Using untapped local genetic resources and the fermented feed under several rearing environments will potentially increase food security and sustainability of the local livestock production (Herrero and Thornton, 2013; Michalk et al., 2019).

In conclusion, this study revealed that different genetic background clearly affects external and internal egg quality traits and amino acid contents and balance of egg yolk and albumen in three chicken genotypes. This study firstly revealed that unique negative phenotypic correlations of amino acids between yolk and albumen in YKD. Further efforts for genetic by environment interaction will give us valuable information for future sustainable livestock production.

## Appendix Supplementary materials

Image, application 1

## Footnotes

Supplementary material associated with this article can be found, in the online version, at doi:10.1016/j.psj.2021.101582.
