# Supplementary material for: Genotype affects free amino acids of egg yolk and albumen in Japanese indigenous breeds and commercial Brown layer chickens
Source: Poult Sci. 2021 Nov 7;101(2):101582. doi: 10.1016/j.psj.2021.101582 (PMC8665412; doi:10.1016/j.psj.2021.101582)
Supplement: Supplementary file 1 [file mmc1.docx]

**Supplementary Table S1.** Statistical results by parametric and non-parametric tests.

| Trait | P_ANOVA | P_Kruskal-wallis test |
| --- | --- | --- |
| EW | 3.031E-15 | 3.25E-08 |
| LLE | 7.602E-12 | 1.76E-07 |
| LSE | 1.027E-11 | 1.24E-07 |
| AW | 1.18E-14 | 1.03E-07 |
| YW | 0.00002992 | 0.0006602 |
| SW | 1.105E-13 | 4.31E-08 |
| ST | 8.389E-10 | 1.26E-06 |
| SCL | 2.2E-16 | 3.48E-07 |
| SCR | 2.2E-16 | 4.76E-08 |
| SCY | 2.2E-16 | 2.70E-07 |
| Y_Asp | 0.008185 | 0.01099 |
| Y_Glu | 0.01631 | 0.01922 |
| Y_Asn | 9.091E-10 | 1.12E-06 |
| Y_Ser | 0.000001375 | 1.02E-05 |
| Y_Gln | 0.000003003 | 1.54E-05 |
| Y_Gly | 6.282E-10 | 6.46E-07 |
| Y_His | 1.8E-11 | 9.83E-07 |
| Y_Arg | 1.226E-09 | 1.04E-06 |
| Y_Thr | 0.002806 | 0.00054 |
| Y_Ala | 1.724E-10 | 2.59E-07 |
| Table S1 (Continued)  Y_Pro | 8.664E-08 | 1.18E-06 |
| Y_GABA | 0.2031 | 0.1592 |
| Y_Tyr | 2.045E-08 | 3.20E-06 |
| Y_Val | 1.883E-09 | 3.71E-07 |
| Y_Met | 3.916E-12 | 2.68E-07 |
| Y_Cys | 1.088E-10 | 4.81E-07 |
| Y_Ile | 0.7921 | 0.8253 |
| Y_Leu | 4.273E-10 | 3.31E-07 |
| Y_Phe | 9.238E-15 | 1.29E-07 |
| Y_Lys | 5.931E-11 | 4.52E-07 |
| A_Asp | 0.00005771 | 7.20E-05 |
| A_Glu | 0.00637 | 0.007054 |
| A_Asn | 0.00432 | 0.009024 |
| A_Ser | 0.03697 | 0.04906 |
| A_Gln | 0.0004754 | 0.001346 |
| A_Gly | 0.0001914 | 0.000256 |
| A_His | 8.184E-13 | 9.03E-07 |
| A_Arg | 5.691E-07 | 1.90E-05 |
| A_Thr | 0.0545 | 0.04206 |
| A_Ala | 0.004821 | 0.004067 |
| A_Pro | 0.09724 | 0.08842 |
| Table S1 (Continued)  A_GABA | 9.193E-09 | 2.73E-06 |
| A_Tyr | 0.871 | 0.7761 |
| A_Val | 0.000001321 | 3.20E-05 |
| A_Met | 0.01279 | 0.004313 |
| A_Cys | 0.01718 | 6.99E-07 |
| A_Ile | 0.0009058 | 0.002193 |
| A_Leu | 0.00002223 | 0.0002956 |
| A_Phe | 0.008101 | 0.01025 |
| A_Lys | 8.545E-10 | 1.01E-06 |
